# Long noncoding RNA LINC01123 promotes the proliferation and invasion of hepatocellular carcinoma cells by modulating the miR-34a-5p/TUFT1 axis

**DOI:** 10.7150/ijbs.45457

**Published:** 2020-06-05

**Authors:** Zunqiang Xiao, Yang Liu, Junjun Zhao, Lijie Li, Linjun Hu, Qiliang Lu, Zhi Zeng, Xin Liu, Dongsheng Huang, Wei Yang, Qiuran Xu

**Affiliations:** 1 The Second Clinical Medical College, Zhejiang Chinese Medical University, Hangzhou, Zhejiang 310053, China.; 2 Key Laboratory of Tumor Molecular Diagnosis and Individualized Medicine of Zhejiang Province, Zhejiang Provincial People's Hospital (People's Hospital of Hangzhou Medical College), Hangzhou, Zhejiang 310014, China; 3 The Medical College of Qindao University, Qindao, Shandong, 266071, China; 4 Graduate Department, BengBu Medical College, BengBu, Anhui 233030, China; 5 Department of Hepatobiliary Surgery, The First Affiliated Hospital of Xi'an Jiaotong University, Xi'an Shaanxi 710061, China

**Keywords:** hepatocellular carcinoma, LINC01123, miR-34a-5p, TUFT1, tumor progression

## Abstract

Hepatocellular carcinoma (HCC), one of the main causes of cancer-related deaths globally, is characterized by rapid growth and high invasiveness. Accumulating evidence demonstrates that long noncoding RNAs (lncRNAs) play critical roles in the growth and metastasis of HCC. Recently, lncRNA LINC01123 has been found to contribute to cell proliferation and aerobic glycolysis in lung cancer. However, the function of LINC01123 in HCC, as well as the underlying mechanism of its action, remain unclear. Here, we found that the expression of LINC01123 was clearly upregulated in HCC tissues compared to nontumor tissues. Furthermore, expression of LINC01123 in HCC cells was significantly higher than in LO2 cells. Importantly, the upregulated level of LINC01123 was related to unfavorable clinical features and poor prognosis of HCC. Next, we demonstrated that LINC01123 knockdown suppressed the proliferation, migration and invasion of HCC cells *in vitro*. Depletion of LINC01123 inhibited HCC xenograft growth* in vivo*. Conversely, ectopic expression of LINC01123 facilitated HCC cell proliferation and invasion. Mechanistically, LINC01123 acted as a molecular sponge for miR-34a-5p in HCC cells. Tuftelin1 (TUFT1) was identified as the target gene of miR-34a-5p. LINC01123 positively regulated TUFT1 level by targeting of miR-34a-5p in HCC cells. Notably, TUFT1 restoration can abolish miR-34a-5p-induced inhibitory effects on HCC cell proliferation, migration and invasion. In conclusion, LINC01123 was overexpressed in HCC and accelerated cancer cell proliferation and invasion by regulating the miR-34a-5p/TUFT1 axis.

## Introduction

Hepatocellular carcinoma (HCC), the most common liver malignancy, is one of the leading causes of cancer-related deaths globally 1. Due to the high rate of infection with hepatitis B virus (HBV), China accounts for half of new cases and deaths of HCC worldwide 2. Although great advances in therapeutic strategies for HCC have been made in recent years, the clinical outcome of patients is still poor due to high frequency of postsurgical recurrence and metastasis 3. Thus, it is crucial to investigate the mechanisms involved in HCC progression and explore new therapeutic targets.

Long noncoding RNAs (lncRNAs), which cannot be translated into proteins and are longer than two hundred nucleotides, have been shown to be key regulators in the initiation and progression of various cancers 4. For example, lncRNA LINC00689 facilitates proliferation, migration, invasion and glycolysis in glioma cells 5. LncRNA IQCJ-SCHIP1-AS1 is expressed at low levels in colorectal cancer (CRC) and inhibits cancer cell proliferation by regulating cell cycle progression and apoptosis 6. It has been widely reported that lncRNAs play critical roles in the regulation of growth and metastasis in HCC by acting as competing endogenous RNAs (ceRNAs) 7-12. For instance, lncRNA MCM3AP-AS1 contributes to the growth of HCC by acting as a molecular sponge for miR-194-5p to enhance forkhead box A1 (FOXA1) expression 7. LncRNA CASC2 represses the migration, invasion and epithelial-to-mesenchymal transition (EMT) of HCC cells by targeting the miR-367/F-box and WD repeat domain containing 7 (FBXW7) axis 8. Upregulation of SNHG7 enhances the proliferation, migration and invasion of HCC cells by sequestering miR-122-5p and increasing ribosomal protein L4 (RPL4) expression 13. As for LINC01123, it has previously been identified as a tumor driver in non-small cell lung cancer (NSCLC) 14. LINC01123, which is transcriptionally activated by c-Myc, facilitates proliferation and aerobic glycolysis in NSCLC cells by targeting the miR-199a-5p/c-Myc pathway 14. Furthermore, LINC01123 combined with three other lncRNAs functions as a promising new prognostic biomarker for head and neck squamous cell carcinoma (HNSCC) 15. However, the functions of LINC01123 in HCC and related mechanisms remain largely unknown.

The current study aims to investigate the expression, biological function and potential mechanism of action of LINC01123 in HCC. Our results indicated high expression of LINC01123 in HCC tissue and cells. LINC01123 accelerated HCC cell proliferation and invasion by targeting the miR-34a-5p/tuftelin1 (TUFT1) axis.

## Material and Methods

### Clinical samples

Eighty pairs of tumor tissues and adjacent nontumor tissues were collected from HCC patients who underwent surgical resection and signed written informed consent at 1^st^ Affiliated Hospital of Xi'an Jiaotong University. None of the participants had received any preoperative treatment. All tissues were pathologically confirmed and immediately preserved in liquid nitrogen. This study was approved by the Ethics Committee of 1^st^ Affiliated Hospital of Xi'an Jiaotong University and complied with the 1964 Helsinki declaration and its later amendments 16.

### Cell culture and transfection

Three human HCC cell lines (HepG2, Huh7 and Hep3B) and a normal human liver cell line (LO2) were maintained in our lab under standard culture conditions 7. The cell lines were passed the test of DNA profiling (STR) in the year of 2018 in the Cell Bank of Type Culture Collection of the Chinese Academy of Sciences (Shanghai, China). A pcDNA3.1/LINC01123 vector was constructed by inserting full-length LINC01123 into the expression vector pcDNA3.1 (OE-1123; Invitrogen, USA). The TUFT1 expression vector (OE-TUFT1) has previously been described 17. The miR-34a-5p mimics/inhibitor and negative control (NC) mimics/inhibitor were purchased from RIBOBIO (Guangzhou, China). LINC01123 siRNA (si-1123-1 and si-1123-2), scrambled siRNA (si-NC), lentivirus-mediated LINC01123 shRNA (sh-1123) and nontargeting shRNA (sh-NC) were obtained from GenePharma (Shanghai, China). Lipofectamine 2000 (Thermo Fisher Scientific, Waltham, MA, USA) was employed for transfection of HCC cells.

### Quantitative real-time PCR (qRT-PCR)

Total RNA was extracted using TRIzol reagent (Thermo Fisher Scientific) and reverse-transcribed with a TIANScript RT Kit (Tiangen Biotech, Beijing, China). Quantitative real-time PCR (qRT-PCR) was performed with a CFX96 Touch™ real-time PCR detection system (Bio-Rad Laboratories, Hercules, CA, USA) using SYBR Green PCR Master Mix (Takara, Shiga, Japan). The relative levels of LINC01123, TUFT1 and miR-34a-5p were calculated with using the 2^-ΔΔCt^ method relative to ACTB and U6. Primer sequences are listed in Supplementary Table 1.

### Cell proliferation assay

For a Cell Counting Kit-8 (CCK-8) assay, 96-well plates were inoculated with transfected HCC cells (5×10^3^ cells/well), and 10 μL CCK-8 solution (Dojindo Laboratories, Dojindo, Japan) was added. Cell viability was measured using a microplate reader (Multiskan™ FC, Thermo Fisher Scientific) at 450 nm. For a 5-ethynyl-2′-deoxyuridine (EdU) assay, transfected HCC cells (5×10^3^ cells/well) were seeded in 96-well plates. Then, the cells were stained using the Cell-Light™ EdU Apollo®488 *In Vitro* Imaging Kit (RIBOBIO). Nuclei were stained with DAPI before being observed with fluorescence microscopy (Olympus, Tokyo, Japan).

### Transwell assays

Transfected HCC cells (1×10^4^) in serum-free medium were added to the upper chamber of transwell inserts coated with Matrigel (BD Biosciences, Franklin Lakes, NJ, USA). The lower chamber was filled with 500 μL of complete medium. After culturing for 24 h, invading cells were fixed with 4% paraformaldehyde and stained with 0.1% crystal violet for imaging under an inverted microscope (Olympus). In addition to the invasion assay, a cell migration assay was conducted without coating with Matrigel.

### Western blot

Total proteins were isolated by RIPA lysis buffer (Beyotime, Shanghai, China) containing PMSF (Beyotime), and total protein concentration was measured with the Bradford protein assay kit (Beyotime). The proteins were separated by 10% SDS-PAGE and transferred onto the PVDF membrane (Millipore, Bedford, MA, USA). Next, membranes were blocked with 5% skimmed milk and incubated (4°C) with primary antibody against TUFT1 (ab184949; Abcam, Cambridge, MA, USA) or GAPDH (sc‑47724; Santa Cruz Biotechnology, Dallas, TX, USA) overnight. Then, membranes were incubated with HRP-conjugated secondary antibodies (Beyotime) and visualized with ECL reagent (Millipore). Blots were imaged using an Amersham Imager 600 (GE Healthcare Life Sciences, Pittsburgh, PA, USA).

### Luciferase reporter assay

Luciferase reporter vectors LINC01123-WT/MT and TUFT1-WT/MT were constructed by inserting the wild-type (WT) LINC01123 or TUFT1 fragment and a LINC01123 or TUFT1 fragment containing mutated (MT) binding sites for miR-34a-5p into pmirGLO expression vectors (Promega, Madison, WI, USA). miR-34a-3p mimics or NC mimics were co-transfected with the above vectors for 48 h in 293T cells. The dual-Luciferase reporter assay system (Promega) was applied for detecting luciferase activity.

### RNA immunoprecipitation (RIP) assay

A RIP assay was performed using the Magna RIP™ RNA-Binding Protein Immunoprecipitation Kit (Millipore), according to the manufacturer's instructions. Briefly, the cultured HCC cells were lysed using RIPA buffer and subsequently incubated with RIP buffer, adding the magnetic bead-bound antibodies against human Ago2 (Proteintech, Rosemont, IL, USA) and normal mouse IgG. Following the total RNA extraction, the precipitated complex was subjected to qRT-PCR.

### *In vivo* tumor growth assay

An *in vivo* tumor growth assay was performed using BALB/C nude mice as previously described 18. Briefly, 1 × 10^6^ Hep3B cell with or without LINC01123 knockdown were injected subcutaneously into nude mice (n=5 per group). Tumor volume was calculated with the formula: tumor volume (mm^3^) = (Long axis × Short axis^2^)/2 every three days. Three weeks after implantation, the xenograft tumor tissues were harvested and subjected to immunohistochemistry for Ki-67 staining 19. The animal studies were approved by the Institutional Animal Care and Use Committee of the Xi'an Jiaotong University.

### Statistical analysis

Data were expressed as the means ± SD (standard deviation) from three independent bio-repeats and analyzed statistically with GraphPad Prism 8.0 (GraphPad Inc., San Diego, CA, USA). Student's *t*-test and one-way analysis of variance (ANOVA) with Tukey's multiple comparison test were used to assess the significance of differences between groups. The survival of HCC patients was analyzed using Kaplan-Meier's method and the log-rank test. Correlation analysis was performed using Pearson's correlation coefficient. The chi-square test was employed to explore the correlations between clinical variables and LINC01123 expression. Univariate and multivariate Cox regression analyses were performed to confirm predictors of overall survival using SPSS version 22 (SPSS, Chicago, IL, USA). An alpha value of P<0.05 was defined to be statistically significant.

## Results

### LINC01123 expression is upregulated in HCC tissues and inversely correlates with patients' survival

**Figure 1:**
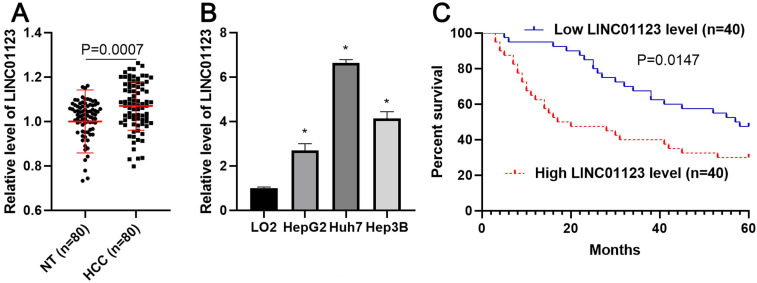
** The expression of LINC01123 is increased in HCC. (A)** The difference in expression of LINC01123 between HCC tissues (n=80) and adjacent nontumor tissues (NT; n=80) was determined with qRT-PCR. LINC01123 expression was related to GAPDH and normalized to the level of LINC01123 in nontumor tissues. **(B)** The expression levels of LINC01123 in the normal hepatic cell line (LO2) and three HCC cell lines (HepG2, Huh7 and Hep3B). **(C)** The “low” or “high” expression of LINC01123 level was defined according to the cut-off value, which was defined as the median value of the cohort of patients tested. The high expression of LINC01123 predicted poor survival of HCC patients. *P<0.05.

**Figure 2:**
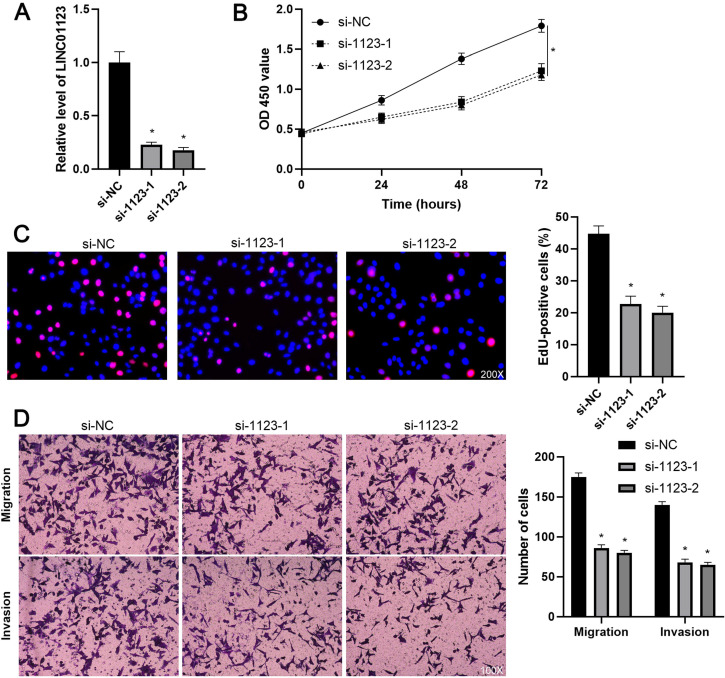
** LINC01123 knockdown represses the proliferation and invasion of Hep3B cells. (A)** Hep3B cells were transfected with control siRNA (si-NC) or LINC01123 siRNAs (si-1123-1 and si-1123-2) and subjected to qRT-PCR for LINC01123 expression. **(B)** CCK-8 assay verified that LINC01123 knockdown inhibited the viability of Hep3B cells. **(C)** Silencing of LINC01123 decreased the percentage of EdU positive Hep3B cells. Original magnification: 200×. **(D)** The numbers of migrating and invading Hep3B cells were reduced by LINC01123 silencing. Original magnification: 100×. *P<0.05.

To clarify whether LINC01123 could act as an oncogene or a tumor suppressor in HCC, qRT-PCR was performed to analyze the expression of LNC01123 in 80 pairs, each consisting of an HCC tissue and a matched tumor-adjacent tissue. We found that LNC01123 expression was significantly upregulated in HCC tissues compared to adjacent nontumor liver tissues (P=0.0007, Figure 1A). Furthermore, we determined the expression of LINC01123 in Huh7, HepG2, Hep3B and LO2 cells. HCC cells expressed higher levels of LINC01123, while LO2 cells expressed lower levels (P<0.05, Figure 2B). Next, the chi-square test found that LINC01123 overexpression was prominently correlated with tumor size ≥ 5 cm (P=0.004), venous infiltration (P=0.025) and advanced TNM stage (P=0.003, Table 1). In addition, the log-rank test indicated that the expression of LINC01123 was also correlated with overall survival (OS) of HCC patients (P=0.0147, Figure 1C). The 5-year OS in the low LINC01123 group was clearly higher than that in the high LINC01123 group (median survival time 57.5 months *versus* 18.5 months). Furthermore, univariate and multivariate Cox regression analysis revealed LINC01123 level to be an independent predictor for indicating OS in HCC patients (P<0.001, Supplementary Table 2). Thus, our data revealed that the upregulated expression of LINC01123 might participate in HCC progression.

### LINC01123 contributes to HCC cell proliferation and invasion

**Figure 3:**
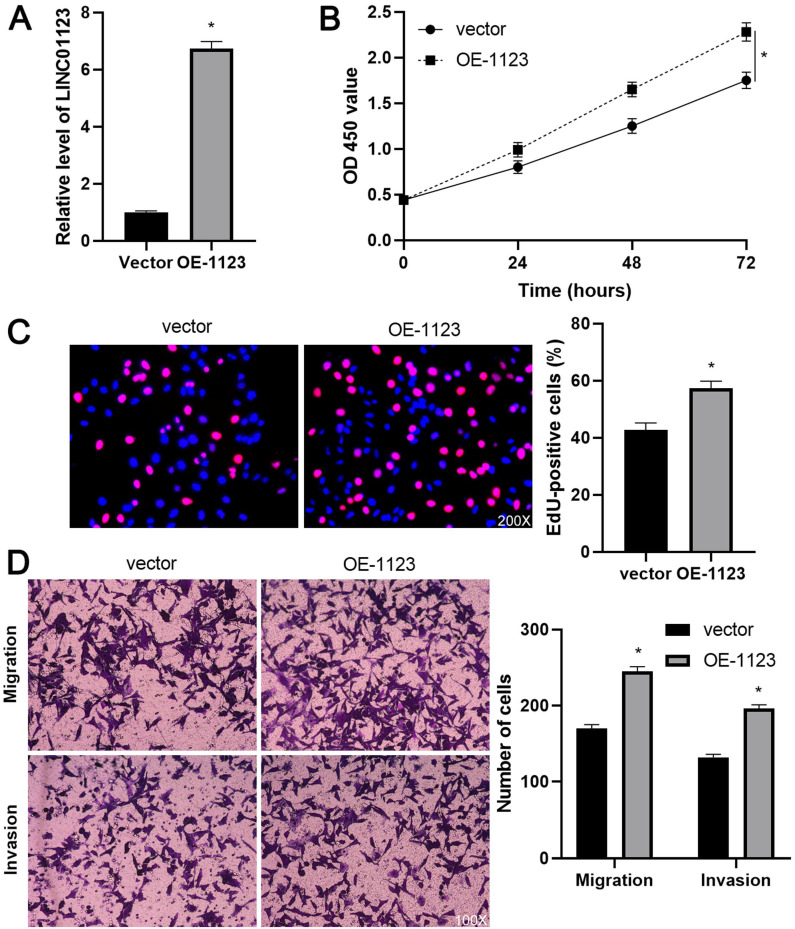
** LINC01123 overexpression promotes Hep3B cell proliferation and invasion. (A)** Hep3B cells were transfected with pcDNA3.1/LINC01123 (OE-1123) or empty vector and measured by qRT-PCR for LINC01123 expression. **(B)** CCK-8 assay demonstrated that LINC01123 overexpression facilitated the viability of Hep3B cells. **(C)** Ectopic expression of LINC01123 increased the percentage of EdU positive Hep3B cells. Original magnification: 200×. **(D)** The numbers of migrating and invading Hep3B cells were increased by LINC01123 overexpression. Original magnification: 100×. *P<0.05.

To elucidate the biological roles of LINC01123 in HCC progression, Hep3B cells were transfected with two different siRNAs to specifically interfere with LINC01123 expression (P<0.05, Figure 2A). These two siRNAs did not affect the expression of NPHP1 mRNA, a genomic neighborhood of LINC01123, in Hep3B cells (Supplementary Figure 1). In the cell proliferation assay, LINC01123 silencing markedly inhibited the proliferation of Hep3B cells (P<0.05, Figure 2B and 2C). Meanwhile, transwell assays revealed that migratory and invasive capacities of Hep3B cells were remarkably reduced by LINC01123 knockdown (P<0.05, Figure 2D). Furthermore, our data consistently showed that LINC01123 depletion markedly repressed the proliferation, migration and invasion of Huh7 cells (Supplementary Figure 2). Next, LINC01123 overexpression was performed in Hep3B and HepG2 cells (P<0.05, Figure 3A and Supplementary Figure 3A). Conversely, to the results from the knockdown assay, ectopic expression of LINC01123 clearly promoted the proliferation, migration and invasion of HCC cells (P<0.05, Figure 3B-3D and Supplementary Figure 3B-3D). Collectively, these results suggest that LINC01123 is a tumor driver of HCC.

### LINC01123 silencing represses tumor growth of HCC *in vivo*

**Figure 4:**
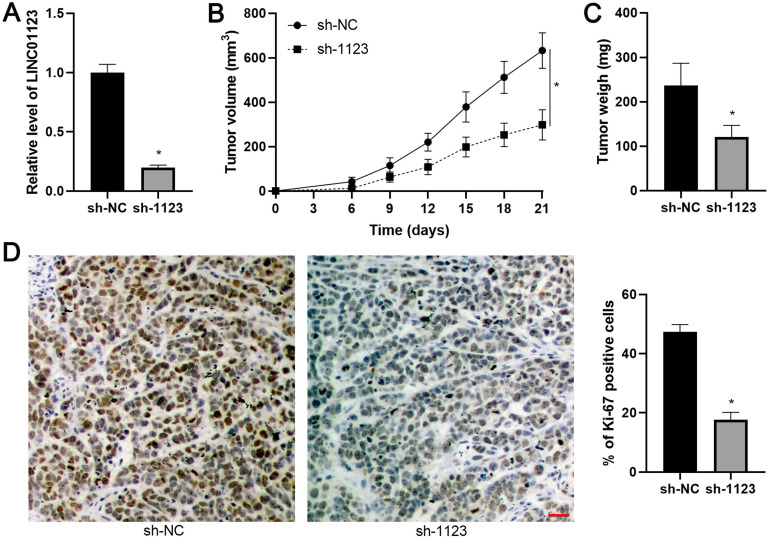
** LINC01123 silencing restrains HCC growth in mice. (A)** Hep3B cells were transfected with negative control shRNA (sh-NC) or LINC01123 shRNA (sh-1123) and subjected to qRT-PCR for LINC01123 expression. **(B) and (C)** Hep3B cells with or without LINC01123 knockdown were subcutaneously injected into nude mice. Both tumor volume and tumor weight in LINC01123 knockdown group (n=5) were obviously lower than those in control group (n=5). **(D)** The percentage of Ki-67 positive cells in tumor tissues from LINC01123 knockdown group (n=5) was obviously lower than that in control group (n=5). Scale bar: 25 μm. *P<0.05.

The effect of LINC01123 on HCC growth *in vivo* was assessed using the subcutaneous xenograft model. LINC01123 knockdown was confirmed by qPCR after the specific shRNA transfection in Hep3B cells (P<0.05, Figure 4A). Smaller tumor volumes and lower tumor weights were observed in the LINC01123 knockdown group compared to the control group, indicating that LINC01123 silencing restrained tumor growth of HCC *in vivo* (P<0.05, Figure 4B and 4C). Moreover, IHC analysis revealed that Ki-67 positive cells in xenograft tissues from the LINC01123 knockdown group were less abundant than in the control group (P<0.05, Figure 4D). Taken together, these *in vivo* data were consistent with *in vitro* results.

### LINC01123 functions as a molecular sponge for miR-34a-5p

**Figure 5:**
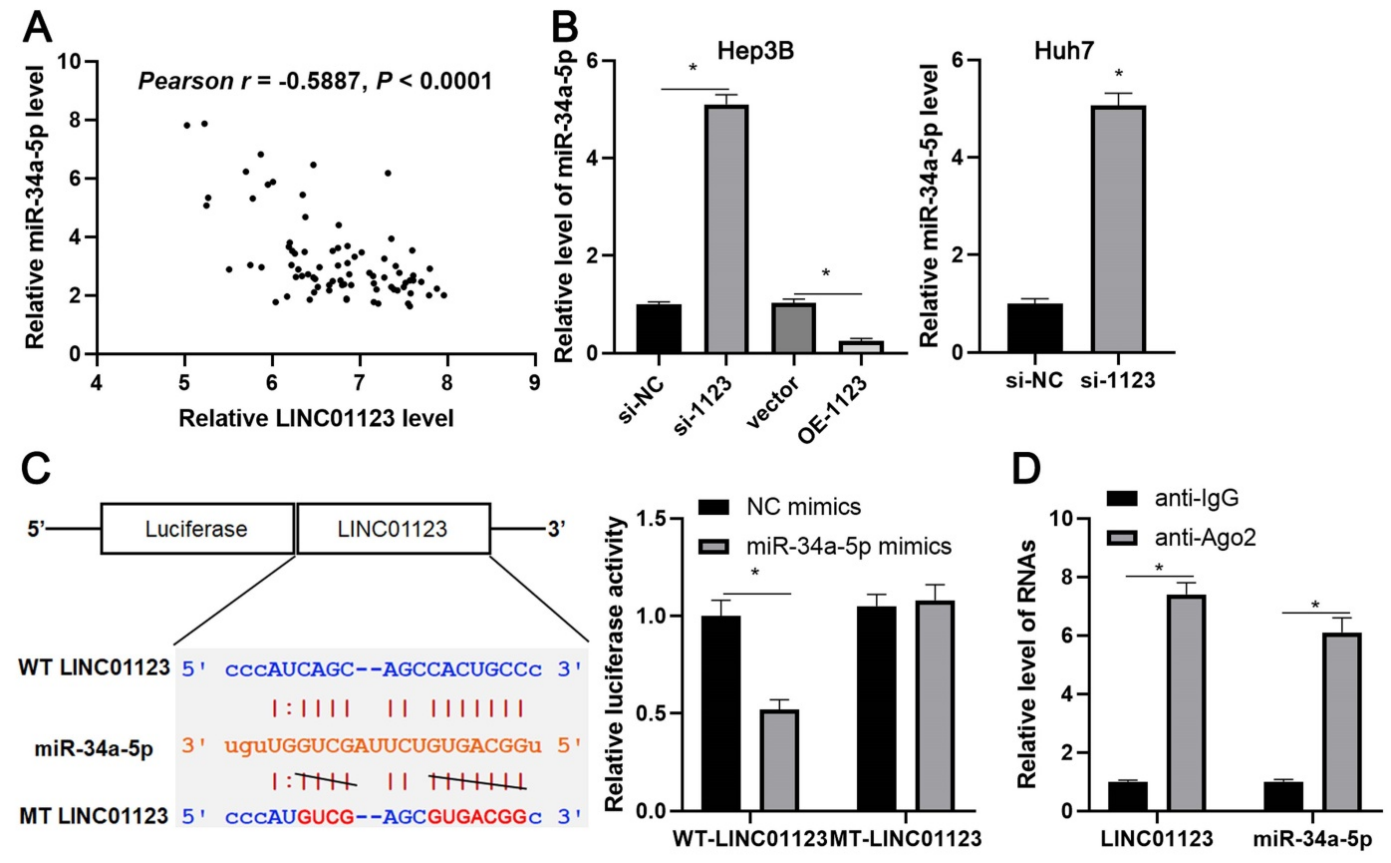
** LINC01123 negatively regulates miR-34a-5p in HCC cells. (A)** The expression of LINC01123 was negatively correlated with miR-34a-5p level in HCC tissues. **(B)** LINC01123 knockdown increased the level of miR-34a-5p in Hep3B and Huh7 cells, whereas LINC01123 overexpression reduced miR-34a-5p expression in Hep3B cells.** (C)** miR-34a-5p overexpression clearly decreased the luciferase activity of vector containing wild type (WT) LINC01123 rather than mutant (MT) LINC01123. **(D)** Enrichment levels of LINC01123 and miR-34a-5p in the Ago2 pellet were significantly higher than those in IgG group. *P<0.05.

Since LINC01123 has previously been reported to act as an miRNA sponge in NSCLC 14, we sought to determine if LINC01123 could sponge corresponding miRNAs to participate in HCC progression. Using starBase V3.0 20, 21, 16 candidate miRNAs were identified. However, only miR-34a-5p was inversely correlated with LINC01123 expression in HCC tissues from the TCGA database (Supplementary Figure 4) and has previously been reported to be a tumor suppressor in HCC 22. Our data also showed an inverse correlation between LINC01123 and miR-34a-5p in HCC tissues (r=-0.5887, P<0.0001, Figure 5A). Interestingly, miR-34a-5p level was negatively regulated by LINC01123 in Hep3B and Huh7 cells (P<0.05, Figure 5B). Next, the dual-luciferase reporter assay and the RIP assay were performed to confirm whether miR-34a-5p is sponged by LINC01123. As expected, miR-34a-5p mimics markedly reduced luciferase activity of the luciferase reporter vector carrying WT-LINC01123 but not MT-LINC01123 (P<0.05, Figure 5C). Furthermore, enriched LINC01123 and miR-34a-5p were detected in the Ago2 pellet compared to the IgG group (P<0.05, Figure 5D). Thus, these data indicated that miR-34a-5p was the target of LINC01123 in HCC cells.

### LINC01123 knockdown inhibits HCC progression by targeting the miR-34a-5p/TUFT1 axis

**Figure 6:**
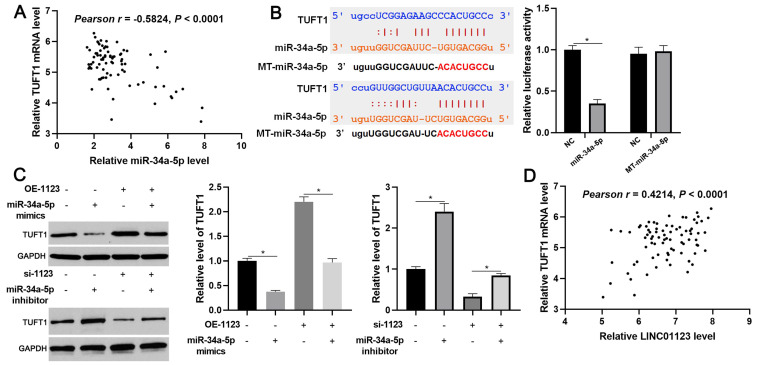
** LINC01123 positively regulated TUFT1 by targeting miR-34a-5p in HCC cells. (A)** The expression of TUFT1 mRNA was negatively correlated with miR-34a-5p level in HCC tissues. **(B)** miR-34a-5p overexpression clearly decreased the luciferase activity of vector containing 3'UTR of TUFT1. However, MT-miR-34a-5p did not affect the luciferase activity of vector carrying 3'UTR of TUFT1. **(C)** Rescue experiment of TUFT1 protein expression by cotransfection of corresponding vectors in Hep3B cells. **(D)** The expression of TUFT1 mRNA was positively correlated with LINC01123 level in HCC tissues. *P<0.05.

**Figure 7:**
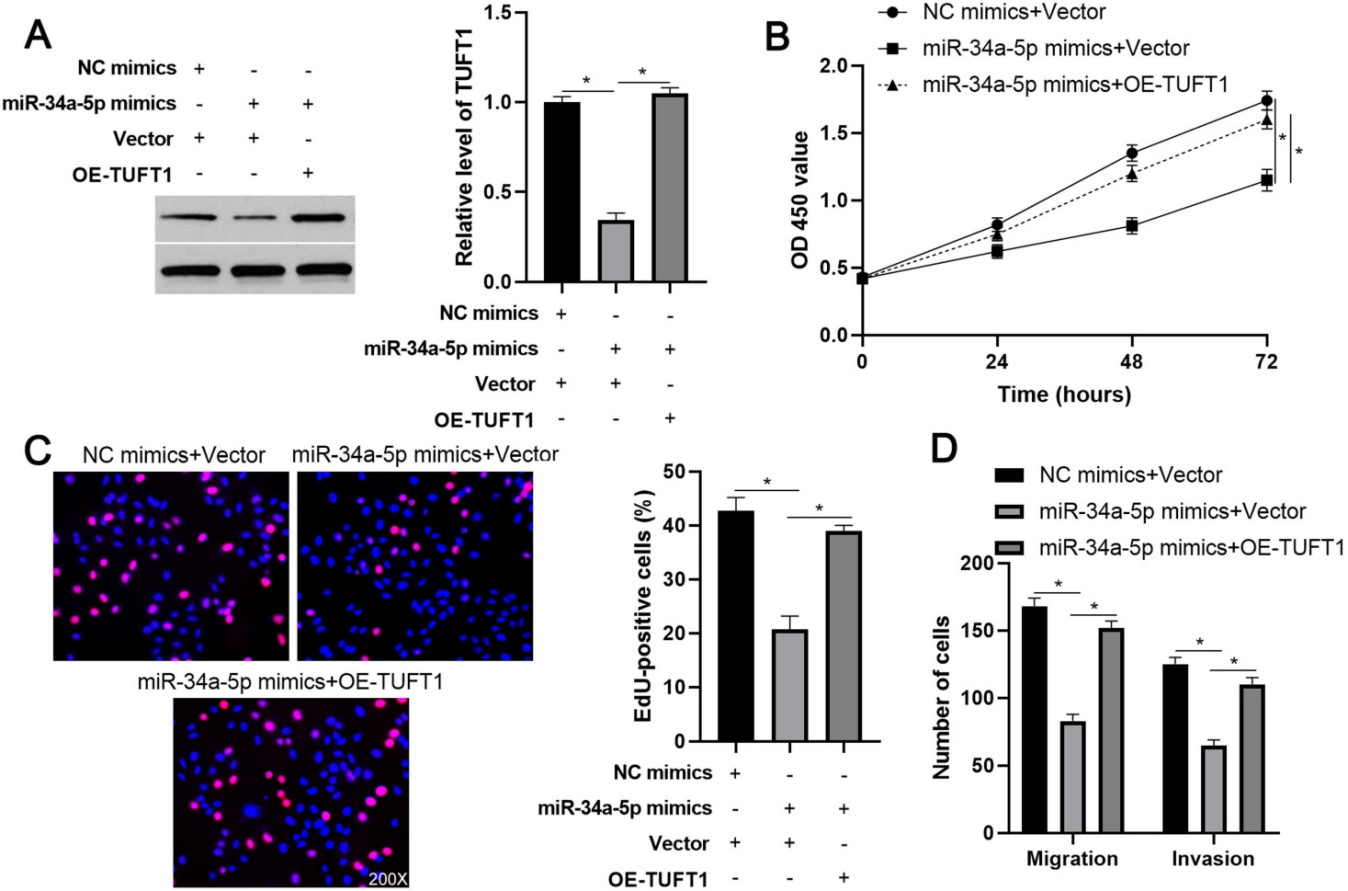
**TUFT1 reverses miR-34a-5p-induced suppressive effects on Hep3B cells. (A)** TUFT1 expression was rescued by transfecting expression plasmid in miR-34a-5p overexpressing Hep3B cells, and Western blot was performed to detect TUFT1 expression. **(B-E)** CCK-8, EdU and transwell assays were carried out to measure proliferation, migration and invasion by Hep3B cells transfected with indicated vectors. Original magnification: 200×. *P<0.05.

We further investigated the downstream mechanism by which miR-34a-5p mediates the function of LINC01123 in HCC. A prediction was made with starBase V3.0 20, 21 to identify putative binding sites of TUFT1 with miR-34a-5p. TCGA data and our data consistently indicated an inverse correlation between miR-34a-5p level and the expression of TUFT1 mRNA in HCC tissues (P<0.0001, Figure 6A and Supplementary Figure 4). Thus, we considered that TUFT1 might be the target of miR-34a-5p. Furthermore, WT-miR-34a-5p, rather than MT-miR-34a-5p, significantly reduced luciferase activity of the luciferase reporter vector containing the 3'UTR of TUFT1 (P<0.05, Figure 6B). In addition, LINC01123 positively regulated TUFT1 abundance by targeting miR-34a-5p in Hep3B cells (P<0.05, Figure 6C). The expression of LINC01123 was positively correlated with TUFT1 mRNA level in HCC tissues (P<0.0001, Figure 6D and Supplementary Figure 4). These results indicate that TUFT1 is a target of miR-34a-5p. Next, TUFT1 restoration was performed by overexpressing miR-34a-5p in Hep3B and Huh7 cells (P<0.05, Figure 7A and Supplementary Figure 5A). Overexpression of miR-34a-5p prominently suppressed proliferation, migration and invasion by Hep3B and Huh7 cells (P<0.05, Figure 7B-7D and Supplementary Figure 5B-5D). TUFT1 restoration significantly reversed the effects of miR-34a-5p on HCC cell proliferation, migration and invasion (P<0.05, Figure 7B-7D and Supplementary Figure 5B-5D). Altogether, our results revealed that LINC01123 can promote HCC progression via the miR-34a-5p/TUFT1 axis.

## Discussion

The aberrantly expressed lncRNAs, which act as diagnostic markers, prognostic indicators or predictors of therapeutic response, have been reported in various types of cancer including HCC 23, 24. For instance, the identification of tumor-specific transcripts (TSTs), which contain many noncoding RNAs and are detectable in extracellular vesicles from HCC patients' blood, may provide new insights into the development of HCC diagnosis 25. Our previous studies have identified MCM3AP-AS1 and A1BG-AS1 as potential biomarkers for predicting the prognosis of HCC 7, 11. In this study, we found LINC01123 expression to be clearly upregulated in HCC tissues. Furthermore, the elevated expression of LINC01123 was correlated with poor prognostic features and reduced OS in HCC patients. Univariate and multivariate analysis revealed that LINC01123 level was an independent predictor of OS in HCC patients. Thus, LINC01123 might predict clinical outcomes in HCC patients.

Solid evidence has suggested that lncRNAs participate in tumor progression by regulating cell proliferation, migration and invasion 26. LncRNA UCA1 facilitates proliferation, migration and invasion by gastric cancer cells and promotes tumor formation and metastasis *in vivo* 27. In addition, lncRNA CRNDE plays an essential role in HCC cell proliferation, migration and invasion 28. A recent study reported that LINC01123 promotes NSCLC cell proliferation and aerobic glycolysis 14. Here, our results indicate that LINC01123 acts as an oncogene by promoting HCC cell proliferation, migration and invasion. *In vivo* experiments further confirmed that LINC01123 knockdown markedly restrained HCC growth.

Since a previous study has verified that LINC01123 functions as a molecular sponge for miR-199a-5p in NSCLC 14, miR-34a-5p was screened as a potential target of LINC01123 using an online platform. miR-34a-5p has been recognized as a tumor suppressor in HCC. Yang P et al*.* demonstrated that miR-34a-5p expression is reduced in HBV-positive HCC, and its suppression facilitates tumor growth and metastasis by enhancing the expression of C-C motif chemokine ligand 22 (CCL22) 22. miR-34a-5p suppresses the migratory and invasive capacities of HCC cells by attenuating c-Met 29. Furthermore, miR-34a-5p overexpression represses the proliferation and induces apoptosis of HCC cells by targeting histone deacetylase 1 (HDAC1) 30. A recent study demonstrates that knockdown of miR-34c-3p inhibited the lactate production, glucose consumption, extracellular acidification rate (ECAR), and aggressive proliferation in HCC cells 31. Previous studies have reported that miR-34a-5p is sponged by lncRNA CCAT2 and lncRNA-MUT 32, 33. The current study revealed that LINC01123 negatively regulates miR-34a-5p level in HCC cells and is inversely correlated with miR-34a-5p expression in HCC tissues. The luciferase reporter assay and the RIP assay further confirmed that miR-34a-5p is the target of LINC01123. Our previous study first identified TUFT1 as a novel oncogene in HCC 17. Moreover, TUFT1 contributes to HCC growth and metastasis by activating Ca^2+^/PI3K/AKT signaling 17. We showed that TUFT1 was a novel target of miR-34a-5p in HCC. Importantly, TUFT1 restoration abrogated miR-34a-5p-induced suppressive effects on HCC cell proliferation, migration and invasion.

In conclusion, we found that LINC01123 is an oncogene in HCC. The upregulated expression of LINC01123 in HCC tissues may potentially predict poor prognosis. LINC01123 accelerated HCC cell proliferation, migration and invasion by sponging miR-34a-5p and enhancing TUFT1 expression. This study may provide new insight into the pathogenesis of HCC.

## Supplementary Material

Supplementary figures and tables.

## Figures and Tables

**Table 1:** Correlation between LINC01123 expression and clinicopathologic characteristics in hepatocellular carcinoma

Characteristics		n=80	LINC01123		*P (chi-square test)*
			Low expression (n=40)	High expression (n=40)	
**Age (years)**	<50	35	17	18	0.822
	≥50	45	23	22	
**Sex**	Male	63	34	29	0.172
	Female	17	6	11	
**HBV**	No	28	16	12	0.348
	Yes	52	24	28	
**Serum AFP level (ng/mL)**	<20	27	15	12	0.478
	≥20	53	25	28	
**Tumor size (cm)**	<5	26	19	7	0.004^*^
	≥5	54	21	33	
**No. of tumor nodules**	1	65	35	30	0.152
	≥2	15	5	10	
**Cirrhosis**	No	35	19	16	0.499
	Yes	45	21	24	
**Venous infiltration**	No	44	27	17	0.025^*^
	Yes	36	13	23	
**Edmondson-Steiner grade**	I+II	57	32	25	0.084
	III+IV	23	8	15	
**TNM stage**	I+II	63	37	26	0.003^*^
	III+IV	17	3	14	

HBV, hepatitis B virus; AFP, alpha-fetoprotein; TNM, tumor-node-metastasis. The “low” or “high” expression of LINC01123 level was defined according to the cut-off value, which was defined as the median value of the cohort of patients tested. *Statistically significant.

## Abbreviations

HCC: hepatocellular carcinoma
HBV: hepatitis B virus
lncRNA: long noncoding RNA
CRC: colorectal cancer
ceRNA: competing endogenous RNA
FOXA1: forkhead box A1
EMT: epithelial-to-mesenchymal transition
FBXW7: F-box and WD repeat domain containing 7
RPL4: ribosomal protein L4
NSCLC: nonsmall cell lung cancer
HNSCC: head and neck squamous cell carcinoma
TUFT1: tuftelin1
TSTs: tumor-specific transcripts
CCL22: C-C motif chemokine ligand 22
HDAC1: histone deacetylase 1
