# Long noncoding RNA LINC01123 promotes the proliferation and invasion of hepatocellular carcinoma cells by modulating the miR-34a-5p/TUFT1 axis: Erratum

**DOI:** 10.7150/ijbs.61069

**Published:** 2021-06-08

**Authors:** Zunqiang Xiao, Yang Liu, Junjun Zhao, Lijie Li, Linjun Hu, Qiliang Lu, Zhi Zeng, Xin Liu, Dongsheng Huang, Wei Yang, Qiuran Xu

**Affiliations:** 1 The Second Clinical Medical College, Zhejiang Chinese Medical University, Hangzhou, Zhejiang 310053, China.; 2 Key Laboratory of Tumor Molecular Diagnosis and Individualized Medicine of Zhejiang Province, Zhejiang Provincial People's Hospital (People's Hospital of Hangzhou Medical College), Hangzhou, Zhejiang 310014, China; 3 The Medical College of Qindao University, Qindao, Shandong, 266071, China; 4 Graduate Department, BengBu Medical College, BengBu, Anhui 233030, China; 5 Department of Hepatobiliary Surgery, The First Affiliated Hospital of Xi'an Jiaotong University, Xi'an Shaanxi 710061, China

In our paper 1, the image of invaded Hep3B cells with empty vector transfection in Figure 3D was mis-pasted. Through repeated confirmation, we captured the images of invaded Hep3B cells with control siRNA or empty vector transfection at the same time and confirmed that we attached the same image of invaded Hep3B cells (control group) in Figure 2D and Figure 3D due to the negligence. Because either control siRNA or empty vector did not change the invasion ability of Hep3B cells, we confirmed that this mistake in Figure 3D did not affect the research results and conclusion of this article. All authors have agreed to the Erratum, and we apologize for the negligence in our work and hope to get the opportunity to correct this mistake. Figure 3D should be corrected as follows.

## Figures and Tables

**Figure 3:**
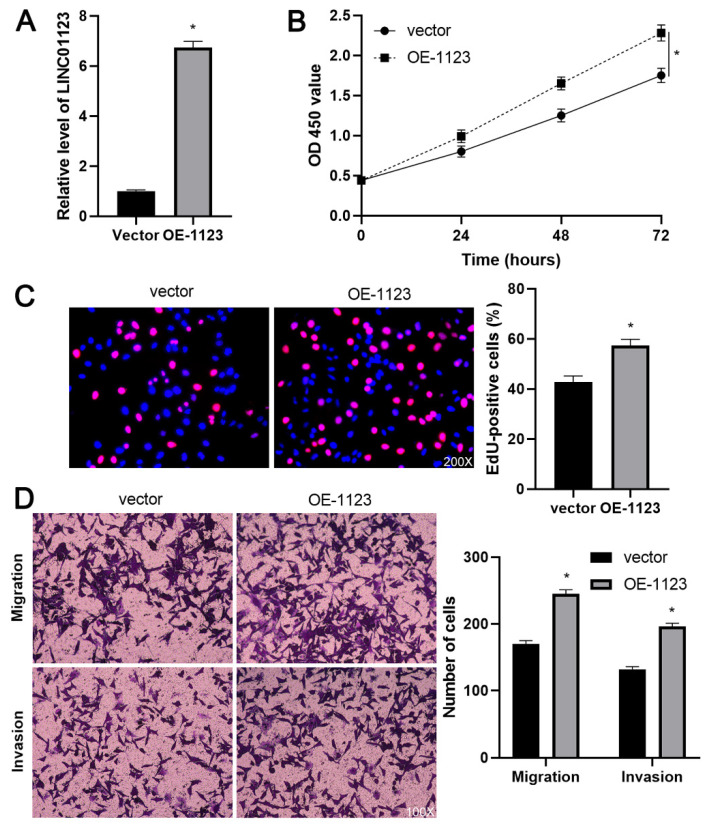
** LINC01123 overexpression promotes Hep3B cell proliferation and invasion.** (A) Hep3B cells were transfected with pcDNA3.1/LINC01123 (OE-1123) or empty vector and measured by qRT-PCR for LINC01123 expression. (B) CCK-8 assay demonstrated that LINC01123 overexpression facilitated the viability of Hep3B cells. (C) Ectopic expression of LINC01123 increased the percentage of EdU positive Hep3B cells. Original magnification: 200×. (D) The numbers of migrating and invading Hep3B cells were increased by LINC01123 overexpression. Original magnification: 100×. *P<0.05.
