# Supplementary material for: Long noncoding RNA LINC01123 promotes the proliferation and invasion of hepatocellular carcinoma cells by modulating the miR-34a-5p/TUFT1 axis
Source: Int J Biol Sci. 2020 Jun 5;16(13):2296–305. doi: 10.7150/ijbs.45457 (PMC7378647; doi:10.7150/ijbs.45457)
Supplement: Supplementary file 1 — Supplementary figures and tables. [file ijbsv16p2296s1.pdf]

Supplementary Table 1. The primer sequences used in this study

Gene	Primer	Sequence (5′-3′)
LINC01123	forward	ACAGTGGCCGCACGCATAGCTG
	reverse	CTGACGACCGAGGTGACAACGATGA
TUFT1	forward	AGAGCCAGCAGCGGAAAGT
	reverse	TTGACTGGATCACAGCTTTTGAA
NPHP1	forward	GTTGGGGCACCTACTGAAGA
	reverse	TGTACATTCCATGCCCTGAA
ACTB	forward	CATGTACGTTGCTATCCAGGC
	reverse	CTCCTTAATGTCACGCACGAT
miR-34a-5p	RT	CTCAACTGGTGTCGTGGAGTCGGCAATTCAGTTGAG CCCCTCTG
	forward	ACACTCCAGCTGTGACTGGTTGACCAGA
	reverse	CTCAACTGGTGTCGTGGA
U6	RT	AACGCTTCACGAATTTGCGT
	forward	CTCGCTTCGGCAGCACA
	reverse	CTCAACTGGTGTCGTGGA

Supplementary Table 2. Univariate and multivariate analysis of factors associated with overall survival of hepatocellular carcinoma patients (n=80)

	Overall survival	
Clinical variables	HR (95%CI)	P value
**Univariate analysis**		
LINC01123 level (high versus low)	3.33 (2.03-5.42)	<0.001*
Sex (female versus male)	0.88 (0.52-1.58)	0.452
Age (≥50 versus <50 years)	0.77 (0.46-1.31)	0.518
Cirrhosis (yes versus no)	1.15 (0.71-1.87)	0.657
Serum AFP level (≥20 versus<20 ng/mL)	1.40 (0.82-2.40)	0.116
Tumor size (≥5cm versus<5 cm)	2.55 (1.60-4.04)	<0.001*
HBV (Yes versus No)	0.95 (0.43-2.13)	0.801
No. of tumor nodules (≥2 versus 1)	1.75 (0.96-3.18)	0.064
Venous infiltration (yes versus no)	3.63 (2.25-5.71)	<0.001*
Edmondson-Steiner grade (III/IV versus I/II)	1.17 (0.69-1.80)	0.567
TNM stage (III/IV versus I/II)	2.82 (1.80-4.57)	<0.001*
**Multivariate analysis**		
LINC01123 level (high versus low)	2.86 (1.74-4.60)	<0.001*
Tumor size (≥5cm versus<5 cm)	2.16 (1.22-3.46)	0.002*
Venous infiltration (yes versus no)	1.98 (1.23-3.08)	0.004*
TNM stage (III/IV versus I/II)	2.12 (1.33-3.70)	0.006*

AFP, alpha-fetoprotein; HBV, hepatitis B virus; TNM, tumor-node-metastasis; HR, hazard ratio; CI, confidence interval.

The "low" or "high" expression of LINC01123 level was defined according to the cut-off value, which was defined as the median value of the cohort of patients tested.

The variables with P-value < 0.05 in the univariate analysis were entered into the multivariate model.

*Statistically significant.

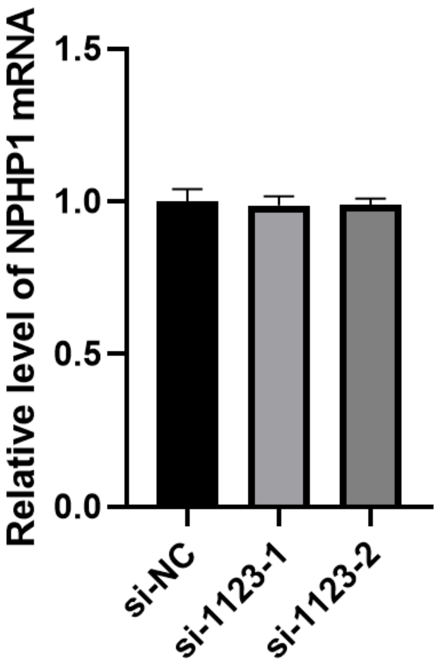

Supplementary Figure 1 LINC01123 siRNAs do not affect the expression of NPHP1 mRNA in Hep3B cells. Hep3B cells were transfected with control siRNA (si-NC) or LINC01123 siRNAs (si-1123-1 and si-1123-2) and subjected to qRT-PCR for NPHP1 expression.

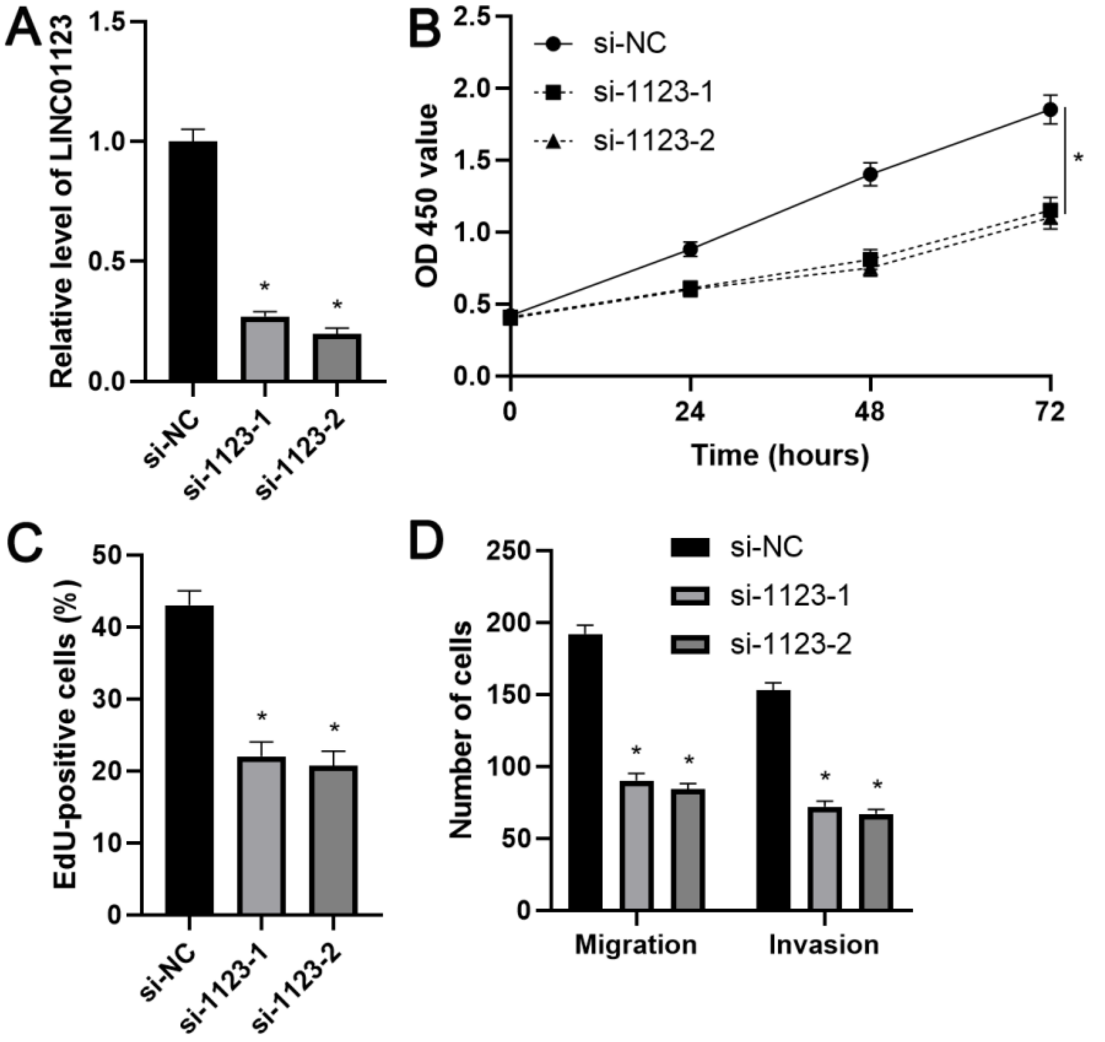

Supplementary Figure 2 LINC01123 knockdown represses the proliferation and invasion of Huh7 cells. (A) Huh7 cells were transfected with control siRNA (si-NC) or LINC01123 siRNAs (si-1123-1 and si-1123-2) and subjected to qRT-PCR for LINC01123 expression. (B) CCK-8 assay verified that LINC01123 knockdown inhibited the viability of Huh7 cells. (C) Silencing of LINC01123 decreased the percentage of EdU positive Huh7 cells. (D) The numbers of migrated and invaded Huh7 cells were reduced by LINC01123 silencing. *P<0.05.

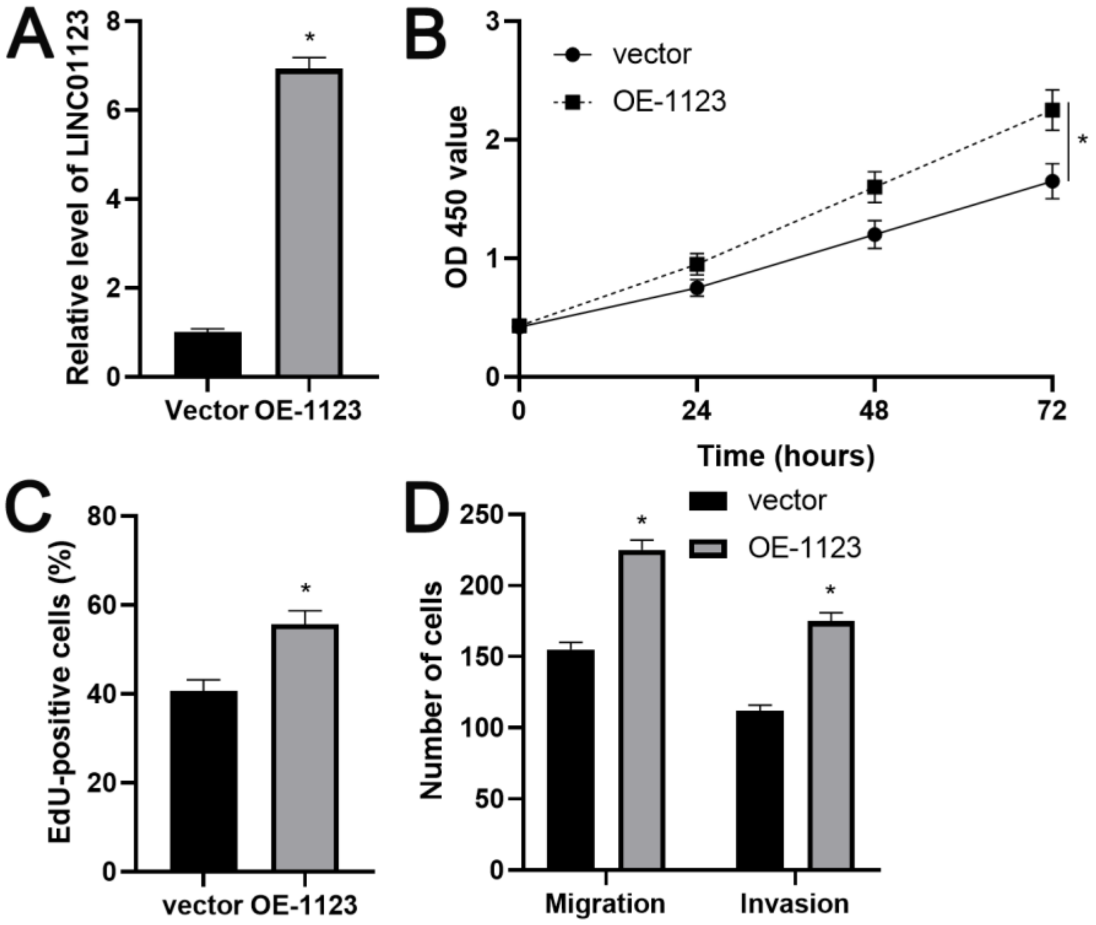

Supplementary Figure 3 LINC01123 overexpression promotes HepG2 cell proliferation and invasion. (A) HepG2 cells were transfected with pcDNA3.1/LINC01123 (OE-1123) or empty vector and measured by qRT-PCR for LINC01123 expression. (B) CCK-8 assay demonstrated that LINC01123 overexpression facilitated the viability of HepG2 cells. (C) Ectopic expression of LINC01123 increased the percentage of EdU positive HepG2 cells. (D) The numbers of migrated and invaded HepG2 cells were increased by LINC01123 overexpression. *P<0.05.

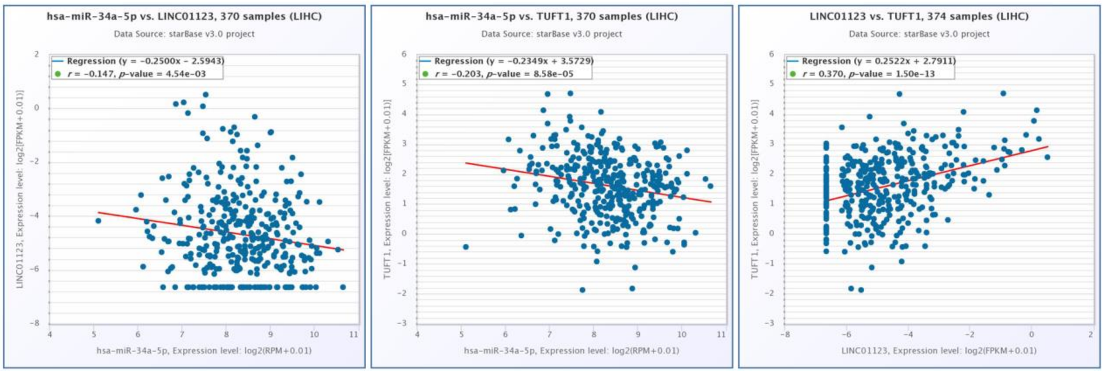

Supplementary Figure 4 The correlations among LINC01123, miR-34a-5p and TUFT1 mRNA in HCC tissues based on TCGA-LIHC data from starBase V3.0 platform

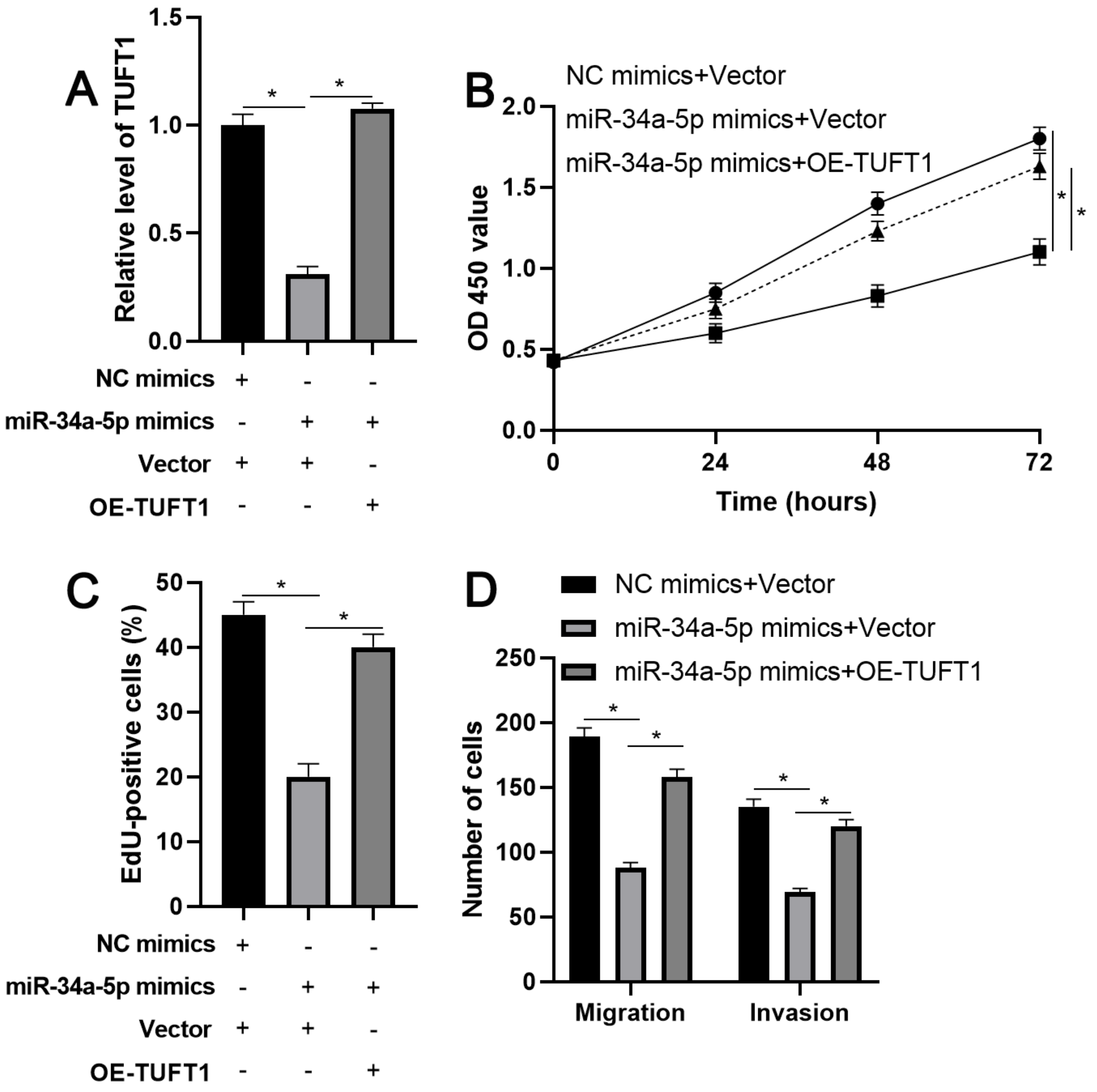

Supplementary Figure 5 TUFT1 abrogates miR-34a-5p-induced inhibitory effects on Huh7 cells. (A) TUFT1 expression was restored by transfecting expression plasmid in miR-34a-5p overexpressing Huh7 cells. (B-D) CCK-8, EdU and transwell assays were carried out to measure the proliferation, migration and invasion of Huh7 cells transfected with indicated vectors. *$P<0.05$.
